# Assessment of apoptosis and autophagy in oral squamous cell carcinoma cell line treated with salivary exosomes

**DOI:** 10.1007/s10266-025-01236-9

**Published:** 2025-11-14

**Authors:** Seham Ahmed Abdel Ghani, Dina Sabry, Amany A. Rabea

**Affiliations:** 1 Oral Pathology, Faculty of Dentistry, Ain Shams University, Cairo, Egypt; 2 Medical Biochemistry and Molecular Biology, Faculty of Medicine, Badr University in Cairo, Cairo University, Giza, Egypt; 3 Oral Biology, Faculty of Oral and Dental Medicine, Future University in Egypt, Cairo, Egypt

**Keywords:** Salivary exosomes, Oral squamous cell carcinoma, Apoptosis, Autophagy

## Abstract

Oral squamous cell carcinoma (OSCC) is a leading cause of cancer-related mortality worldwide. Salivary exosomes (sExosomes), a subtype of extracellular vesicles, have gained attention for their role in intercellular communication and their potential as non-invasive diagnostic and therapeutic tools. This study investigated the therapeutic effects of sExosomes on OSCC by examining cellular ultrastructure and assessing the expression of caspase-3 and the autophagy-related gene NKX2-3. Human OSCC cells (SCC-25) were cultivated in Dulbecco’s Modified Eagle Medium (DMEM) containing 10% exosome-depleted fetal bovine serum (FBS) and 1% penicillin–streptomycin (PS). sExosomes were isolated from human saliva using differential ultracentrifugation and confirmed via transmission electron microscopy (TEM). The experiment included two groups: Group I (untreated OSCC cells) and Group II (OSCC cells treated with sExosomes for 14 days). Ultrastructural examination revealed abnormal mitotic figures, chromatin clumping, and enlarged mitochondria in Group I, whereas Group II showed features characteristic of apoptosis, including pyknotic nuclei, cytoplasmic vacuolization, and degenerated organelles. ELISA results indicated significantly higher caspase-3 levels in Group II (305.33) compared to Group I (91.03), suggesting enhanced apoptotic activity. Conversely, NKX2-3 expression was significantly lower in Group II (0.89) than in Group I (2.65), indicating suppression of autophagy-related signaling. Statistical analysis was conducted using an independent-samples t-test, with significance set at p < 0.05. These findings support the potential of sExosomes to modulate tumor behavior by promoting apoptosis and inhibiting autophagy in OSCC, underscoring their promise as novel therapeutic agents in future cancer treatment strategies.

## Introduction

Oral squamous cell carcinoma (OSCC) is one of the primary causes of cancer-related deaths worldwide, with a particularly high prevalence in both developing and developed countries. In 2019 alone, approximately 5300 Americans were diagnosed with OSCC, resulting in nearly 10,800 fatalities [1]. Despite considerable progress in therapeutic interventions, the five-year survival rate for OSCC persists below 60%, with only minimal improvement over the past two decades. The poor prognosis is primarily attributed to late-stage diagnoses, highlighting the urgent need for earlier, more effective diagnostic methods to improve treatment outcomes and overall patient survival [2, 3].

One such promising diagnostic approach is liquid biopsy, a non-invasive method for detecting cancer biomarkers in biofluids. Originally focused on blood, liquid biopsy has expanded to include saliva, urine, and cerebrospinal fluid, each offering distinct advantages in terms of accessibility and ease of collection [4]. Among these, saliva is particularly valuable due to its non-invasive collection, its vital role in oral and digestive health, and the presence of various biomarkers associated with systemic health [5, 6]. Furthermore, the antimicrobial properties of saliva, attributed to components such as immunoglobulin A, lactoferrin, and lactoperoxidase, enhance its potential as a diagnostic medium for OSCC [7–9]. Salivary diagnostics has advanced rapidly, owing to the rich molecular information contained in saliva, which can reflect both oral and systemic health status [10].

Exosomes, a subtype of extracellular vesicles (EVs), play a significant role in cell communication and have garnered attention as promising biomarkers for OSCC and other oral diseases [11, 12]. These small vesicles, which originate from cellular membranes and are secreted into the extracellular space, transport a range of molecular cargo, including proteins, nucleic acids, and metabolites [13]. Exosomes are involved in several biological processes, such as immune regulation, stromal remodeling, and tumor metastasis. In the context of OSCC, exosomes have been identified as potential biomarkers for early detection and disease monitoring, as well as vehicles for targeted molecular delivery, thus offering considerable promise for improving patient management [14].

A critical aspect of OSCC progression is the dysregulation of apoptotic pathways. Apoptosis, also known as programmed cell death, is a critical mechanism for maintaining cellular homeostasis by selectively eliminating damaged or defective cells [15–17]. However, the failure of apoptosis mechanisms can lead to the survival of malignant cells, promoting tumor development, metastasis, and resistance to treatment [18–21]. Caspase-3, an executioner caspase in the apoptotic cascade, is central to this process, causing cellular breakdown by cleaving key substrates, including DNA repair proteins [22]. The activation of caspase-3, which occurs via both intrinsic and extrinsic apoptotic signals, is a hallmark of apoptotic cell death and has significant implications for cancer therapy [23–27]. In addition to apoptosis, autophagy, a mechanism of programmed cell death, exerts a dual role in cancer, often allowing tumor cells to survive under metabolic stress and resist therapies [28–30]. Autophagy inhibitors are being explored as potential treatments to sensitize tumor cells to conventional therapies [31, 32]. In OSCC, dysregulated autophagy is linked to poor prognosis, with autophagy-related genes (ARGs) emerging as potential therapeutic targets [33, 34].

NKX2-3, a transcription factor implicated in multiple cellular processes, including cell proliferation, metabolism, and immune responses, has also been implicated in OSCC and other cancers [35, 36]. Recent research suggests that NKX2-3 regulates key pathways, such as the Wnt signaling cascade, and could serve as an early biomarker for cancer detection and treatment [37, 38]. Despite its potential, NKX2-3's role in OSCC remains underexplored, particularly in relation to apoptosis and exosome-mediated tumor progression.

This study seeks to investigate the role of sExosomes in OSCC progression by examining their effects on caspase-3 activation and NKX2-3 expression. Through the analysis of ultrastructural changes in exosome-treated cells, we seek to provide novel insights into the molecular mechanisms underlying OSCC and to assess the therapeutic potential of exosome-based biomarkers in enhancing treatment strategies.

## Materials and methods

### Ethical approval

This study was conducted in accordance with the ethical guidelines approved by the Research Ethics Committee of Ain Shams University (FDASU-Rec ER042521). No informed consent is required.

### Study design and sample collection

This experimental study included two groups:

- *Group I*: Non-treated OSCC cells
- *Group II*: sExosomes—treated OSCC cells

Purposive sampling was employed to select human saliva samples from healthy adult volunteers aged 18–40 years. A total of 20 saliva samples were collected and equally distributed between the two groups. Participants included in the study were healthy individuals without any history of oral diseases, systemic illnesses, or recent antibiotic use. Individuals presenting with oral infections, a history of smoking, or any diagnosed systemic conditions were excluded to minimize confounding variables that could affect the composition and integrity of sExosomes.

### Cell culture

The human oral squamous cell carcinoma cell line SCC-25 was procured from VACSERA Holding Company, Egypt. Cells were sustained in DMEM enriched with 10% bovine calf serum (Gibco BRL) and 1% PS, incubated at 37 °C under 5% CO₂. Upon reaching 80–90% confluency, the cells were trypsinized, reseeded, and subcultured every 2–3 days. For assays, 3–5 × 10^5^ cells were plated per well in 6-well plates [39, 40].

### sExosomes cultivation

sExosomes were obtained either directly from human saliva or from cultured salivary gland epithelial cells and oral cell lines. Cells were maintained in DMEM fortified with exosome-depleted FBS to prevent contamination. Cultures were incubated at 37 °C in 5% CO₂ until reaching approximately 70–80% confluency (5 × 10⁶ cells/ml). The medium was then replaced with fresh exosome-depleted media, and cells were incubated for an additional period of 24–48 h to promote exosome secretion [41, 42].

### sExosomes isolation and characterization

Saliva samples underwent sequential centrifugation steps to remove cellular debris and larger vesicles:

- 300 × g and 2000 × g to eliminate cells and debris
- 10,000 × g to remove apoptotic fragments and large extracellular vesicles

Samples were subsequently passed through a 0.22 µm membrane to filter out residual impurities. Exosomes were isolated via ultracentrifugation at 100,000 × g for 70–120 min. Alternatively, precipitation-based kits and size-exclusion chromatography methods were employed as appropriate. TEM was used to confirm exosome presence by visualizing their characteristic cup-shaped morphology [43].

### Treatment procedure

After reaching 80–90% confluency, OSCC cells in Group II were treated with isolated sExosomes, while Group I remained untreated. The treatment period extended for 14 days, with regular observation.

### TEM preparation

For TEM analysis, cells were centrifuged at 2000 × g for 10 min and preserved using 3% glutaraldehyde (in 0.1 M sodium cacodylate buffer, pH 7.0) for 2 h at room temperature. After rinsing, cells underwent secondary fixation with 1% osmium tetroxide for 2 h. Dehydration was conducted through an ethanol gradient (10–100%), then immersed for 30 min in absolute ethanol. Specimens were infiltrated with epoxy resin/acetone mixtures and embedded within pure resin. Ultrathin sections were mounted on copper grids and subsequently stained with uranyl acetate and lead citrate before TEM examination [44–46].

### Caspase-3 assay: apoptosis assessment

Apoptosis levels were assessed via ELISA measuring caspase-3 activity. Treated and control cells were lysed to extract intracellular proteins, then added to plates pre-coated with cleaved caspase-3 antibodies. A secondary enzyme-linked antibody was applied, followed by a substrate that produced a measurable color change. Absorbance was detected using a spectrophotometer, with higher readings indicating greater apoptosis levels [47].

### Autophagy-related gene expression via ELISA

Autophagy was evaluated by quantifying NKX2-3 protein expression using a specific ELISA kit. After treatment under autophagy-inducing conditions, cells were lysed and applied to plates coated with antibodies against NKX2-3. Standard ELISA protocol was followed, and absorbance was measured spectrophotometrically. Results reflected relative NKX2-3 expression and autophagy activation [48].

### Statistical analysis

Data analysis was performed using the Statistical Package for the Social Sciences (SPSS), version 26.0 (SPSS Inc., Chicago, IL, USA). Quantitative data were expressed as mean ± standard deviation (SD), along with their respective ranges. An independent-samples t-test was conducted to analyze the differences between the two groups. A 95% confidence interval was applied with a 5% margin of error. Statistical significance was established at a p-value threshold of less than 0.05.The required sample size per group was estimated using the following formula for comparing two independent groups: n = 2 × (Zα/2 + Zβ) 2 × σ2 / Δ2. Where Zα/2 = 1.96 for a 95% confidence level, Zβ = 0.84 for 80% power, σ is the estimated standard deviation of the caspase-3 activity or NKX2-3 protein expression and Δ is the expected effect size (Cohen’s d). Due to the limited availability of prior studies reporting effect size values for sExosomes treatment in OSCC cell lines, a moderate effect size of Cohen’s d ≈ 0.7 was assumed based on conventions in biological research and supported by Lakens (2013) [49]. This estimation will be refined once pilot data for caspase-3 activity is available. Additionally, in the most relevant study examining the modulation of OSCC by sExosomes, no specific Δ (delta) or effect size values were reported, while they documented significant molecular changes in miR‑1307‑5p expression and its association with disease aggressiveness and chemoresistance, quantitative effect size measures were not provided [50].

## Results

### Characterization of sExosomes

**Fig. 1:**
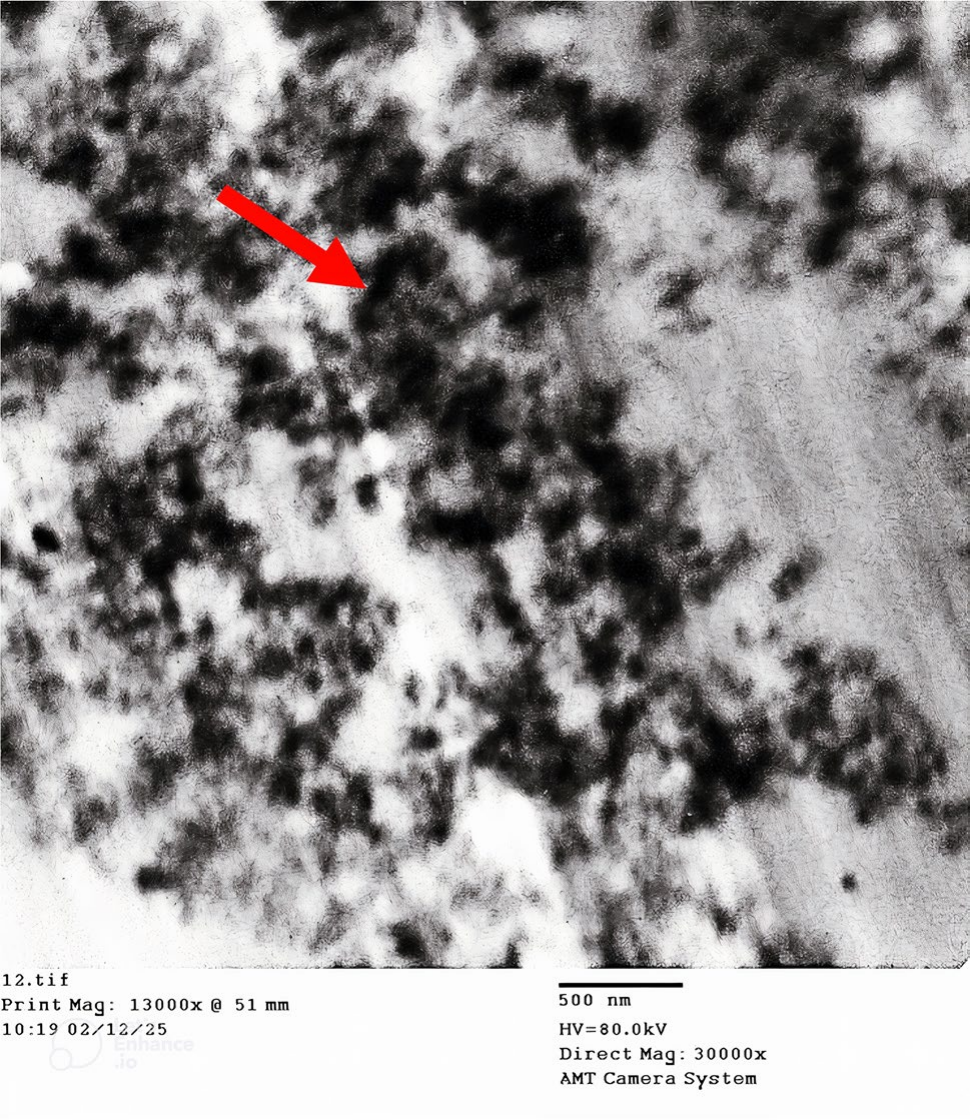
A photomicrograph of sExosomes shows them as variably sized, oval-shaped, electron-dense particles (red arrow)

TEM confirmed the presence of sExosomes as oval, electron-dense vesicles with heterogeneous sizes, consistent with the typical morphology of exosomes. These vesicles exhibited a lipid bilayer membrane and ranged in diameter from approximately 30–150 nm (Fig. 1).

### Ultrastructural findings

#### Group I (untreated OSCC cells)

**Fig. 2:**
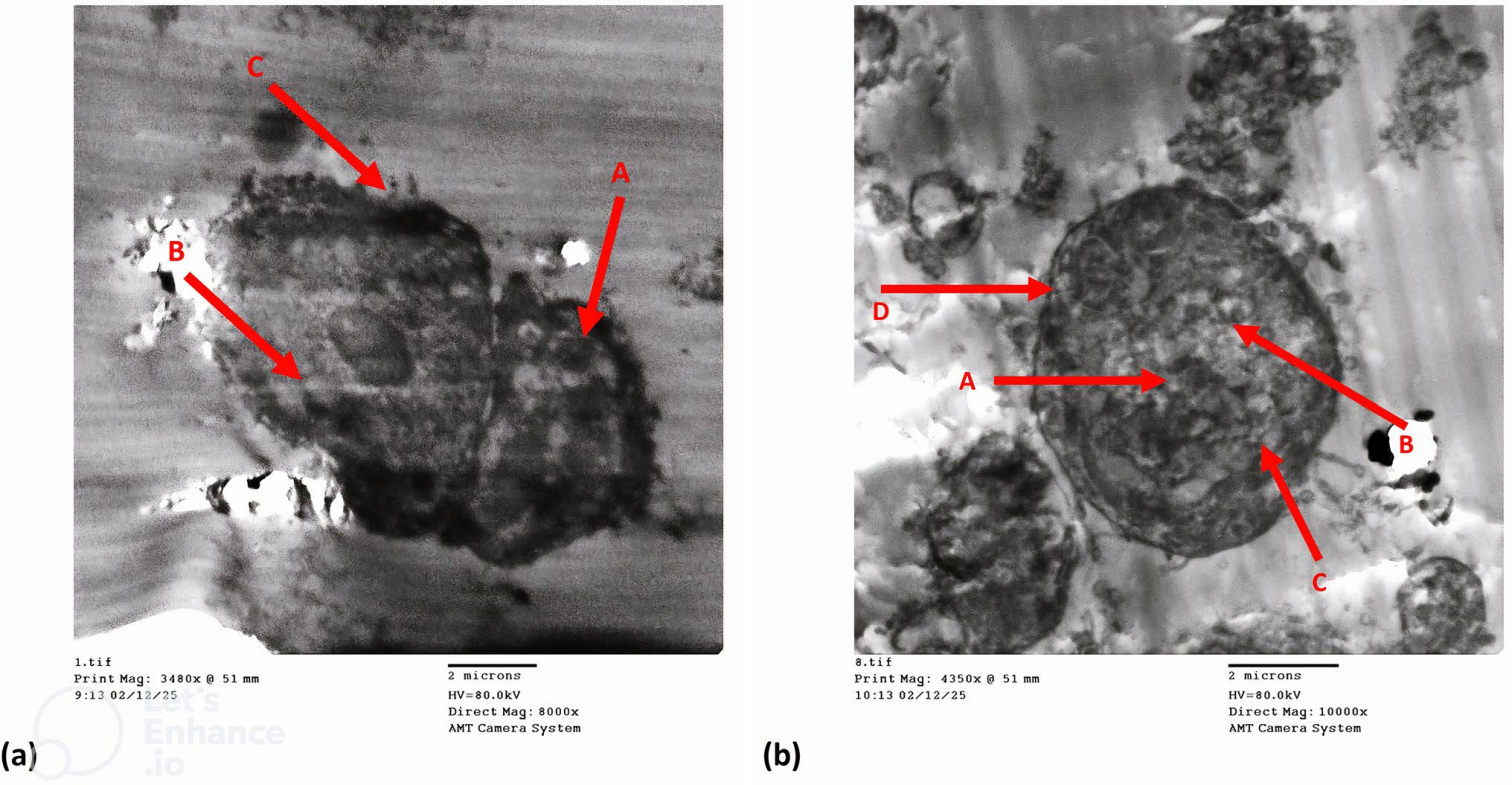
Transmission electron micrographs of **a-** Group I showing abnormal mitotic figures (A), apparently large sized mitochondria (B) and microvilli of cytoplasmic membrane (C). **b-** Group II showing pyknotic nucleus (A), degenerated mitochondria and other cellular organelles (B), cytoplasmic vacuoles (C) as well as regular cell membrane (D)

TEM examination revealed classical hallmarks of malignancy. Cancer cells exhibited abnormal mitotic figures, such as chromosome clumping and disorganized mitotic spindles. Additionally, mitochondria were markedly enlarged, and dense, prominent microvilli were observed on the cell membrane, indicative of active cellular proliferation (Fig. 2a).

#### Group II (OSCC cells treated with sExosomes)

Exosome-treated OSCC cells displayed significant ultrastructural changes suggestive of apoptotic and degenerative processes. These included pyknotic nuclei, degenerated mitochondria, disrupted cytoplasmic organelles, and prominent cytoplasmic vacuolization. Despite these intracellular changes, the integrity of the plasma membrane appeared to be preserved (Fig. 2b).

### Statistical analysis

#### Caspase-3 expression

**Fig. 3:**
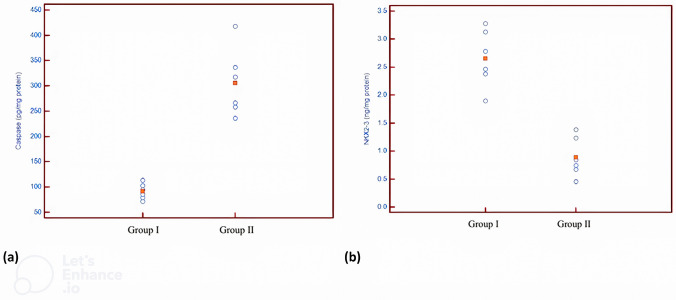
Dot plot of statistical results illustrating mean values of: caspase (pg/mg protein) in all groups **(a)**, NKX2-3 (ng/mg protein) in all groups **(b)**

ELISA analysis of caspase-3 expression revealed a statistically significant increase in Group II compared to Group I (p < 0.001), indicating enhanced apoptotic activity in sExosome-treated cells. (Table 1, Fig. 3a).

**Table 1:** Comparison between Group I and Group II with respect to caspase 3 levels (pg/mg protein)

Caspase 3	Mean	± SD	± SE	Min	Max	t–test	p-value
Group I	91.03	15.56	6.35	71.60	113.40		
Group II	305.33	66.67	27.22	235.70	417.60	− 7.668	0.001*

Analysis was conducted using an independent samples t-test for Mean ± SD

*SD* refers to standard deviation, *SE* to standard error, *Min* to the minimum value, and *Max* to the maximum value

^**^p-value < 0.001 indicates a highly significant difference

#### NKX2-3 expression

Analysis of the autophagy-related marker NKX2-3 demonstrated a highly significant increase in expression levels in Group II relative to Group I (p < 0.001), reflecting a notable modulation of autophagy pathways following sExosome treatment (Table 2, Fig. 3b).

**Table 2:** Comparison of NKX2-3 levels (ng/mg protein) between Group I and Group II

NKX2-3	Mean	± SD	± SE	Min	Max	t–test	p-value
Group I	2.65	0.51	0.21	1.90	3.28		
Group II	0.89	0.35	0.14	0.45	1.39	6.938	0.001*

An independent samples t-test was used to analyze the data presented as Mean ± SD

*SD* Standard deviation, *SE* Standard error, *Min* Minimum, *Max* Maximum

^**^p-value < 0.001 denotes a highly significant result

#### Sample size distribution

Summary statistics for Caspase-3 and NKX2-3 levels, including sample size (n = 6 per group), mean and SD are presented in (Table 3).

**Table 3:** Sample size distribution, Caspase 3 and NKX2-3 (ng/mg protein) levels in Group I and Group II

Group	n	Caspase-3 Mean ± SD (pg/mg)	NKX2-3 Mean ± SD (ng/mg)
Group I	6	91.03 ± 15.56	2.65 ± 0.51
Group II	6	305.33 ± 66.67	0.89 ± 0.35

An independent samples t–test was employed to analyze the data presented as Mean ± SD; n; sample size number

## Discussion

Oral squamous cell carcinoma (OSCC) ranks as the sixth most prevalent malignant tumor worldwide, primarily affecting the lips, tongue, and buccal mucosa [51]. Despite advances in diagnosis and therapy, OSCC continues to pose significant clinical challenges due to its aggressive nature and poor prognosis. Chemotherapy remains a primary treatment modality for invasive cancers; however, its efficacy is often limited by drug resistance and undesirable side effects [52]. Therefore, identifying novel therapeutic agents that offer enhanced efficacy with reduced toxicity is of paramount importance.

Recent studies have highlighted the pivotal role of exosomes in modulating various cancer-related processes, including immune evasion, chemotherapeutic resistance, tumor progression, angiogenesis, and metastasis [53]. Exosomal contents, such as proteins, lipids, DNA, mRNA, and non-coding RNAs, act as bioactive molecules capable of mediating intercellular communication within the tumor microenvironment. In this context, exosomes have emerged as both potential biomarkers and therapeutic agents, particularly in liquid biopsy approaches for OSCC.

Apoptosis, or programmed cell death, is a crucial regulatory process in cancer development and therapy. The dysregulation of apoptotic pathways contributes to tumor progression and resistance to treatment. Apoptosis-related proteins have been proposed as biomarkers for prognosis and as therapeutic targets [54]. The landmark study by Hanahan and Weinberg [55] identified the ability of cancer cells to evade apoptosis as one of the key hallmarks of cancer. Apoptosis is tightly regulated by a balance between pro-apoptotic factors such as Bax and caspases, and anti-apoptotic factors including Bcl-2, Bcl-xL, mutant p53, and survivin [56, 57]. Caspases, especially caspase-3, play a central role in the execution phase of apoptosis, and their expression is frequently used as an indicator of apoptotic activity in various cancers [58, 59].

In OSCC, p53 mutations and downregulation of caspase-3 expression contribute to the inhibition of apoptosis, resulting in unchecked cell proliferation and malignant transformation [60, 61]. Apoptosis is morphologically characterized by nuclear fragmentation, chromatin condensation, and DNA fragmentation into nucleosomal units of approximately 180–200 base pairs by endogenous endonucleases [62, 63]. In the current study, cancer cells treated with sExosomes displayed hallmark morphological changes consistent with apoptosis, including pyknotic nuclei and cytoplasmic vacuolization. These observations are consistent with the results of Qing et al. [64], who demonstrated apoptotic features in cervical adenocarcinoma and SCC cells treated with exosomes.

Our results demonstrated significantly lower caspase-3 expression in Group I (untreated OSCC cells) compared to Group II (sExosome-treated cells), suggesting enhanced apoptosis in the latter. Reduced caspase-3 expression may contribute to tumor cell resistance against microenvironmental stress [65]. sExosomes may promote apoptosis through delivery of pro-apoptotic molecules that facilitate mitochondrial membrane permeabilization, triggering the intrinsic apoptotic pathway. This mechanism aligns with previous studies showing that therapeutic agents like zebularine induce apoptosis in SCC-9 and SCC-25 cells via caspase-3 and PARP-dependent pathways [66–68]. A study on MDA-MB-231 breast cancer cells further supported this by demonstrating increased PARP cleavage and caspase-3 activation following treatment [69].

Autophagy, a cellular degradation process, also plays a complex role in tumorigenesis. It can function as a tumor suppressor by removing damaged organelles and inhibiting inflammation, but under certain conditions, it may promote tumor survival and progression [70, 71]. Autophagy also influences tumor response to therapeutic interventions [72]. In head and neck squamous cell carcinoma (HNSCC), autophagy is implicated in both tumor development and therapy resistance, underscoring the need for deeper mechanistic insights [73]. Despite this, specific studies investigating autophagy-related mechanisms in OSCC remain limited [34].

In the present study, NKX2-3 expression was significantly higher in Group I than in Group II, suggesting that sExosomes downregulate NKX2-3, potentially disrupting tumor-promoting autophagy signaling. These findings are supported by Kerkhofs et al. [36], who linked NKX2-3 to key biological functions such as angiogenesis, immune regulation, metabolism, and cellular proliferation. Overexpression of NKX2-3 has been associated with OSCC pathogenesis, particularly through suppression of apoptotic signaling pathways such as Wnt. It is proposed that sExosomes inhibit NKX2-3 expression by degrading its mRNA transcripts, thereby restoring apoptotic potential in cancer cells [74].

NKX2-3, a member of the NKX homeobox gene family, plays a role in tumor initiation and progression. It is implicated in the activation of oncogenic pathways such as B-cell receptor signaling, NF-κB, and PI3K/AKT [75, 76]. NKX2-3 is predominantly expressed in the nucleus of tumor cells and has been proposed as a predictive biomarker for response to FOLFOX4 chemotherapy [77]. Previous studies have also identified NKX2-3 as a prognostic marker in HNSCC [78, 79].

Our study is the first to use sExosomes in OSCC therapy; up until now, they have mostly been used for diagnostic purposes. Previous research has primarily focused on identifying salivary exosomal biomarkers for the early detection and prognosis of OSCC. For instance, Gai et al. (2018) [80] identified differentially expressed miRNAs in salivary extracellular vesicles from OSCC patients, while He et al. (2020) [81] demonstrated the diagnostic value of salivary exosomal miR-24-3p. Nakamichi et al. (2021) [82] reported elevated levels of the Alix protein in sExosomes as a potential biomarker, and Patel et al. (2022) [50] found that miR-1307-5p in sExosomes correlates with tumor aggressiveness and chemoresistance. Unlike these studies, which focused on biomarker identification and disease monitoring, our work uniquely positions sExosomes as therapeutic agents, marking a novel shift from diagnosis to treatment in the management of OSCC.

However, a major limitation of our study in using sExosomes for OSCC treatment is the insufficient understanding of their precise therapeutic mechanisms. Furthermore, factors such as variability in exosome content, inconsistent isolation and purification methods, and concerns about their stability and targeted delivery pose challenges to clinical application. Most existing research is confined to in vitro studies, making it difficult to predict in vivo effectiveness and safety. Additionally, small sample sizes and the absence of long-term safety data limit the reliability and generalizability of current results.

## Conclusion

The findings of this investigation suggest that sExosomes hold potential as a therapeutic strategy for OSCC.Their ability to upregulate caspase-3 expression, promoting apoptosis, and downregulate the autophagy-related gene NKX2-3, which is associated with tumor progression, underscores their potential in modulating critical molecular pathways involved in carcinogenesis. These dual effects indicate that sExosomes could serve as effective molecular tools in OSCC treatment. Further comprehensive studies are warranted to elucidate the underlying mechanisms and validate their clinical applicability, which may ultimately contribute to the development of innovative and less toxic cancer therapies.

## Abbreviations

OSCC: Oral squamous cell carcinoma
SD: Standard deviation
P-value: Probability value
